# The value of fractional exhaled nitric oxide in occupational diseases – a systematic review

**DOI:** 10.1186/s12995-022-00355-1

**Published:** 2022-07-25

**Authors:** Marina Ruxandra Oțelea, Anne Kristin M. Fell, Claudia Mariana Handra, Mathias Holm, Francesca Larese Filon, Dragan Mijakovski, Jordan Minov, Andreea Mutu, Euripides Stephanou, Zara Ann Stokholm, Sasho Stoleski, Vivi Schlünssen

**Affiliations:** 1 Carol Davila University of Medicine and Pharmacy, Bucharest, Romania; 2 Occupational and Environmental Medicine, Telemark Hospital, Skien, Norway; 3 Department of Global Health and Community Medicine, Institute of Health and Community, University of Oslo, Oslo, Norway; 4 Colentina Clinical Hospital, Clinic for Occupational Medicine, Bucharest, Romania; 5 Occupational and Environmental Medicine, School of Public Health and Community Medicine, Institute of Medicine, Sahlgrenska Academy, University of Gothenburg, Gothenburg, Sweden; 6 Unit of Occupational Medicine, Department of Medical Sciences, University of Trieste, Trieste, Italy; 7 Institute of Occupational Health of RN Macedonia, Skopje, North Macedonia; 8 Faculty of Medicine, Ss. Cyril and Methodius, University in Skopje, Skopje, North Macedonia; 9 Central Military University Emergency Hospital “Carol Davila”, Bucharest, Romania; 10 University of Crete, Heraklion, Greece; 11 Department of Occupational Medicine, Danish Ramazzini Centre, Aarhus University Hospital, Aarhus, Denmark; 12 Department of Public Health, Environment, Occupation and Health, Danish Ramazzini Centre, Aarhus University, Aarhus, Denmark

**Keywords:** Fractional exhaled nitric oxide (FeNO), Occupational asthma, Occupational bronchitis, Occupational interstitial lung disease, Occupational hypersensitivity pneumonitis

## Abstract

Fractional exhaled nitric oxide (FeNO) is a non-invasive biomarker of respiratory tract inflammation, originally designated to identify eosinophilic airway inflammation and to predict steroid response. The main field of application of this biomarker is asthma, but FeNO has also been used for other allergic and non-allergic pulmonary disorders such as chronic obstructive pulmonary disease, hypersensitivity pneumonitis and interstitial lung disease. A substantial part of respiratory diseases are related to work, and FeNO, a safe and easy measure to conduct, is a potential valid examination in an occupational setting.

This systematic review assesses the value of measuring FeNO related to three types of airborne exposures: allergens, irritants, and respiratory particles inhaled during occupational activities. The review covers results from longitudinal and observational clinical studies, and highlights the added value of this biomarker in monitoring effects of exposure and in the diagnostic criteria of occupational diseases. This review also covers the possible significance of FeNO as an indicator of the efficacy of interventions to prevent work-related respiratory diseases.

Initially, 246 articles were identified in PUBMED and SCOPUS. Duplicates and articles which covered results from the general population, symptoms (not disease) related to work, non-occupational diseases, and case reports were excluded. Finally, 39 articles contributed to this review, which led to the following conclusions:

a) For occupational asthma there is no consensus on the significant value of FeNO for diagnosis, or on the magnitude of change needed after specific inhalation test or occupational exposure at the workplace. There is some consensus for the optimal time to measure FeNO after exposure, mainly after 24 h, and FeNO proved to be more sensitive than spirometry in measuring the result of an intervention. b) For other occupational obstructive respiratory diseases, current data suggests performing the measurement after the work shift. c) For interstitial lung disease, the evaluation of the alveolar component of NO is probably the most suitable.

**Supplementary Information:**

The online version contains supplementary material available at 10.1186/s12995-022-00355-1.

## Introduction

Three decades ago Alving, Weitzberg and Lundberg [1] reported the relation between asthma, eosinophilic inflammation, and the fractional exhaled nitric oxide (FeNO) level. Still numerous unresolved questions related to the utilization of this test persist. Mainly associated with the eosinophilic inflammation and allergy, FeNO has been particularly related to asthma, asthma-COPD overlap syndrome [2], some forms of COPD [2], acute and chronic eosinophilic pneumonia, bronchiolitis obliterans syndrome, and allergic rhinitis [3].

The use of FeNO for the identification of Th2 inflammation in asthma has been extensively studied, but controversies continue even on this topic: while the Global Initiative for Asthma (GINA) does not recommend the inclusion of FeNO in the decision guidance for treatment in asthma [4] the joint statement of the European Respiratory Society and the American Thoracic Society [5] and several national position papers recommend to include FeNO [6, 7] in the diagnostic of certain phenotypes of asthma. In the context of interstitial lung diseases, alveolar concentration of NO was associated with the extension of interstitial fibrosis, fibroblast proliferation, and lung functional parameters [8], but there is no consensus to recommend it for diagnosis, prognosis, or treatment decisions.

Work related respiratory diseases cover a broad range of respiratory illnesses. In fact, the population attributable fraction of occupational non-malignant respiratory diseases varies from low (1% for tuberculosis) to intermediate (16% for asthma and 30% for other granulomatous diseases including sarcoidosis) [9, 10]. Measuring FeNO in persons exposed to respiratory hazards has many advantages: it is a non-invasive, simple to perform test, not costly and can be used in outpatient clinics. In well-selected populations, FeNO can help differentiating asthma from other respiratory disorders in primary care [11].

In view of the advantages described above and the fact that the respiratory tract represents an important route for a multitude of occupational hazards, FeNO has a potential important role in occupational medicine practice.

Through this systematic review, we aim to assess the value of using FeNO in occupational diseases.

## Methods

Our specific study question was: Is there evidence for using FeNO in the diagnosis of occupational lung diseases?

We performed a systematic search in PUBMED and SCOPUS databases using specific search terms. “FeNO” and “occupation” were the mandatory keywords, to which we added one of the following terms: “asthma”, “occupational bronchitis”, “hypersensitivity pneumonitis (HP)”, “fibrosis”, ‘pneumoconiosis” “allergens, “particles”, “dust”, “vapours”, “gazes”, “fumes” or “chemicals”. We did not limit the selection of the articles by year of publication.

### Description of the exposure

Papers describing persons exposed to allergens, chemicals, particles (inorganic or organic dust) in the working environment were included, and so were papers on patients with occupational respiratory diseases. In the final analysis, only articles containing a description of the exposure (type of occupational hazard, industry, process) or a comprehensive evaluation of exposure (e.g. for regulatory purposes, such as conducted in clinics were patients were referred to for confirmation of an occupational disease) were used.

### Type of comparators

A comparison between exposed, less exposed, or nonexposed persons was used to support the results.

### Outcomes

Occupational asthma (OA), other occupational obstructive lung diseases, and occupational interstitial lung disease were the outcomes, which were considered for this systematic review. Under the term of “other occupational lung diseases” we have included both the occupational bronchitis and the occupational chronic obstructive pulmonary disease (COPD). The interstitial lung disease covered also the pneumoconiosis.

### Inclusion and exclusion criteria

Peer review articles on humans (cohort, case-control, cross-sectional) that included FeNO measurement(s) in relation with a defined occupational disease were included.

The following exclusion criteria were adapted:

1. Articles in which the outcome was a work-related respiratory symptom and not an occupational or a work-related disease
2. Reviews of any type, case presentations, conference papers, editorials, short letters, and commentaries
3. Animal studies or cellular experiments
4. Articles referring to exposure in the general population or without specific content on occupational exposure
5. Non-English papers

### Data extraction and processing

Studies were searched until February 2021.The extracted files were divided between all co-authors and classified according to the primary endpoint, population, number of participants, occupational hazards, industries, occupations, comparators, and main results. The authors were also asked to describe the strengths and the weaknesses of the studies, which served for grading them in the final step.

After this first round of screening, the articles were, grouped in 3 categories which reflected the purpose of this systematic review: 1) asthma, 2) other obstructive lung disease, and 3) interstitial lung disease. The last one included the pneumoconioses and occupational HP.

### Quality assessment

The study design, recruitment strategy, exposure assessment, handling of confounding factors, and adjustment for covariates were used to assess the quality of the studies, following the methodology described by the Effective Public Health Practice Project (EPHPP) quality assessment tool for quantitative studies [12]. In the appreciation of selection bias, we followed sample representativeness according to scope: for example, if more than 90% of workers in the specific workplace were included, we graded the recruitment strategy as strong, even if for the total number of people working in the industry was small. We are aware that the results of such a “strong” ranked study are not necessarily strong enough for a guideline definition, but they are strong enough to support the initiation of larger trials about the use of FeNO in a specific industry, exposure, or disease. In studies conducted in reference centers for the diagnosis of occupational diseases the selection bias is somehow inevitable;,as they are selected by the referring physician. Therefore, they were classified as “somewhat likely to be representative” and included in the “moderate” rating.

The specific confounding factors for FeNO testing were considered to be the following: age, height, body mass index (BMI), sex, diurnal variation, smoking, acute infectious diseases and nitrate-containing foods [13, 14].

Usage of validated questionnaires or standardized methods were mandatory to grade as strong data collection. In particular, for the FeNO measurement, compliance with the recommendations of the American Thoracic Society [15] was necessary for inclusion in this category. This implies the measurement of FeNO at expiratory flow of 50 ml/s. As it is the most widely used techique, it will be further mentioned as FeNO.

The blindness was appreciated as strong only if clear blindness about the scope of the study for both investigators and subjects, which is very rarely the case in occupational medicine research because explaining the study to the participants and their already knowledge about the occupational exposure prevents blindness.

## Results

**Fig. 1:**
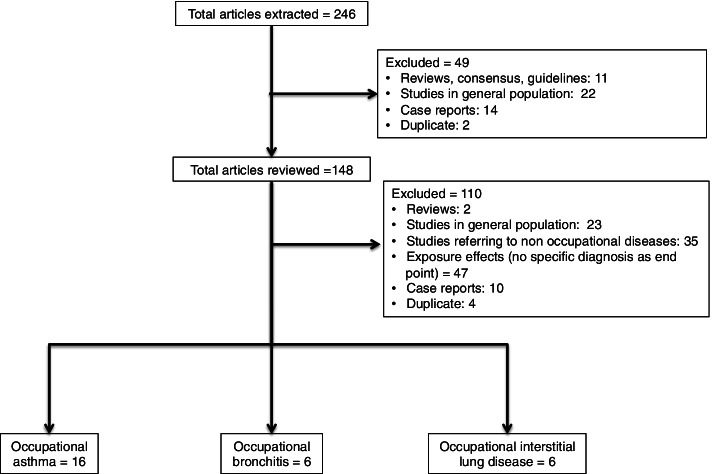
Selection process for the reviewed articles

In the initial stage, 246 articles were identified from the databases. After the automatically filtering for reviews, case studies and age of participants (to exclude studies on children and adolescents), 148 were reviewed. Among these, the title and abstract screening identified 23 studies referring to the general population, 35 analyzing non-occupational diseases, 10 case reports and case series with less than 15 participants, and 3 duplicates. Thirty-six studies referred only to symptoms, without a targeted diagnosis and 11 publications analyzed only the exposure effect of various allergens. This led to 28 articles which were included in the final analysis (Fig. 1).

### FeNO utilization for occupational asthma

In total, 16 studies had as primary endpoint the utilization of FeNO for asthma. Of these, 13 addressed the value of FeNO in the diagnostic criteria, and 3 studies included FeNO as a surrogate endpoint for an intervention.

#### Utilization of FeNO as criterion for diagnosing OA

The identification of a significant threshold value of FeNO - either the baseline value or the variation after workplace exposure or specific inhalation challenge test (SIC) - was the main purpose of these studies. In most cases, they referred to the variation of FeNO from baseline to 24 h after a SIC, but there were also a few studies which considered the variation of FeNO compared to the reference values in the general population [16].

Based on the subjects included in the studies, we identified two different designs (See Supplementary Table 1, Additional File 1): the first included research conducted in specialized centers to confirm the suspicion of OA in patients with very diverse exposure.

The second type of study design focused on a specific exposure (agent or occupation) or a category of OA agents, categorized in high molecular weight (HMW) and low molecular weight (LMW).

Six studies conducted in reference centers used SIC as a confirmation test for OA and one the serial peak expiratory flow (PEF) monitoring. The exposure assessment was comprehensive, as generally requested by the specific compensation rules in each country. They considered possible confounders and used standard methods for the measurement of all variables (See Supplementary Table 2, Additional File 2).

Overall, the reference center studies showed that FeNO after SIC increased significantly compared with before SIC, but reduced sensitivity of the final expert based diagnosis of OA [17, 18]. The relation with SIC depended on the level considered as a significant variation of FeNO before and post challenged [17–19], the presence of atopy [18] and on the type of agent (HMW versus LMW) [20]. There was no agreement on the optimal FeNO variation; the cutoff varied from ≥10 ppb to 17.5 ppb, 25 ppb, and even 50 ppb. The sensitivity ranged between 36.8 and 45.3% and the specificity between 81.2 and 100%.

One study used as a reference test the serial PEF measurement analyzed by Oasys computer program [21]. For smokers with higher than 14.7 ppb and non-smokers with higher than 22.1 ppb FeNO values measured within 24 h of work exposure had good correlation with the nonspecific bronchial hyper-responsiveness.

Van Kampen et al. [22] conducted a small study to measure FeNO for 2 weeks of work exposure and 2 weeks without work exposure. A cutoff of 20 ppb was set as a significant work-related increase. Patients underwent a comprehensive evaluation (atopy, agent specific sensitization, lung function and serial FEV_1_, nonspecific and specific bronchial hyperresponsiveness) and were finally classified as OA or non-OA by a medical expert. Based on the 20 ppb cutoff, nine out of ten finally classified as OA showed an increase of FeNO after work exposure. All positive FeNO cases came from exposure to substances known to induce immunologic OA. Four out of 23 cases which were finally classified as non-OA did not show a 20 ppb increase: one with cobalt exposure, one exposed to formaldehyde and plastic dust, one to lacquers, and one to detergent enzymes. Except for the last one, all the others showed no sensitization to the incriminated agent.

Atopy increased the baseline level of FeNO [19]. Inconsistent results concerning the FeNO variation after SIC were found in different samples [18, 23]. Exposure to HMW agents was the only factor associated with a ≥ 17 ppb variation in FeNO after SIC [23] in a large sample of OA diagnosed by SIC. In another study, the baseline FeNO value was higher in HMW than LMW agent exposure, but a significant increase post SIC was found only for LMW agents [19]. A relatively small study dedicated to LMW agents found no significant differences in FeNO change (increase by 20% or > 6 ppb) 24 h after SIC in 16 positive compared to 16 negative SIC cases [24]. To sum up, it is difficult to draw any conclusion about the significance of FeNO variation related to the molecular weight of the occupational agents, partly because the threshold considered as significant was different; in some studies, the number was too small to reach the statistical significance and, furthermore, the final assessment as OA differs in each country.

The studies dedicated to some specific exposures generally include a low number of cases. When SIC was available, the significance of baseline FeNO or variation of FeNO was compared to this gold standard. For example, in the case of cleaning products, such as sodium hypochlorite [25], a significant increase was found after SIC with bleach, but this was not reflected in all SIC positive cases. In another study which included patients exposed to a mixture of cleaning products [26] the baseline FeNO values were similar to those in the control group.

SIC with isocyanates induced a significant increase in FeNO in two independent studies aiming to identify the mechanism of inflammation in this type of asthma [27, 28]. The first identified the airway wall as the source of the FeNO increase. After 24 h, both FeNO at expiratory flow of 50 ml/s and the bronchial FeNO concentration increased significantly only in the SIC positive patients. The second found a good correlation between the variation of FeNO and sputum eosinophils and provided interesting data on the duration of the increase in FeNO after SIC, which peaked at 24 h only in the SIC positive patients. Levels higher than the initial ones were maintained up to 7 days after SIC, even if not statistically significant.

We found only one study referring to bakers and hairdressers, two occupations exposed to a variety of allergens, most of which are in the HMW category,. Although the number of persons included in the analysis was rather large, the number of cases was low and the imbalance between these two occupations in cases and controls could bias the exposure [29]. As in the studies conducted in reference centers, there was a better specificity than sensitivity of the baseline FeNO values for asthma diagnosis, with a cutoff set to > 25 ppb, as recommended by the American Thoracic Society [15]. When the levels of FeNO were compared to the theoretical reference values for age, gender, and smoking status [16], or when the cutoff was set to lower levels (> 8.5 ppb) together with a positive questionnaire, the specificity decreased while the sensitivity increased [29]. A positive questionnaire was considered if the person reported a diagnosed asthma or at least one of the respiratory symptoms (wheezing, breathlessness, chest tightness, cough and sputum) during the last 12 months; the symptoms have appeared after inception of apprenticeship; and symptoms are present during the working days and improve or disappear during week-ends or holidays [30].

#### Efficacy of an intervention

FeNO was also used as a surrogate endpoint for the efficacy of an intervention in the prevention of respiratory symptoms and OA (Table 1).

**Table 1:** Studies in which FeNO was utilized as surrogate endpoint of an intervention in a workplace with potential risk of occupational asthma.

	Exposure/occupation	Comparator	No pf participants	Type of intervention	Main results
Al Badri F.M. et al.,2020 [31]	Flour/bakers	2 types of interventions vs no intervention	149 dust control intervention + training/ 162 training/ and 113 controls	exposure reduction and education	1. Significant decrease in FeNO (*p* = 0.24) is associated with reduction in exposure if baseline FeNO > 25 ppb
					2. Workers with work-related ocular-nasal symptoms at baseline, had a significant decline in FeNO (≥ 10 ppb) in the intervention group.
Dressel H. et al., 2007 [32]	Cow dander and storage mites	intervention vs no intervention	81 exposed/24 controls	education	1. In the intervention group, FeNO decreased significantly (*p* < 0.05)
					2. In the intervention group, the decrease was higher in subjects with baseline elevated FeNO > 35 ppb (*p* < 0.01). 3. Spirometric values did not changed significantly in the two groups.
Dressel H. et al., 2009 [33]	Cow dander and storage mites	intervention vs no intervention	43 exposed/ 15 controls	education	1. The decrease in FeNO was maintained 1 year after the intervention(*p* = .001); in the control group there was a slight but not statistically significant increase in FeNO.
					2. Spirometric values did not changed significantly in both groups.

One study [31] compared two interventions (education + better exposure control) with an educational program alone and with no intervention in a randomized group trial. The study concerned 18 supermarket bakeries in one town and the group randomization process referred to a selection of an equal number of units stratified based on the number of employees and production output. The outcomes of the intervention consisted of a reduction of the work-related respiratory symptoms and a more than 10% decrease of the initial FeNO. A year after the intervention, a reduction was observed only in subjects with an initial FeNO > 25 ppb. No other objective measure (e.g. bronchial hyperresponsiveness or lung function) was used to compare the effectiveness of the intervention.

Another project, conducted by Dressel et al. was dedicated to the prevention of respiratory symptoms and allergies in animal farmers [32] by introduction of new educational program. This study included only farmers with diagnosed occupational asthma. FeNO and lung function were measured before and after the program implementation. Particularly in those with high initial values, FeNO was reduced. The achieved low FeNO values were maintained after another year of follow up [33]. Spirometric values did not changed significantly neither in the short term (4–6 weeks) after the interevntion, nor after 1 year. The selection bias and changes in the exposure management during the follow up [31], or the dropout rate [33] were the elements which classified 2 out of the 3 studies in the group with moderate quality (Table 2).

**Table 2:** FeNO as surrogate endpoint of an intervention in prevention of OA: grading of the studies

Study	Selection bias	Type of study	Confounders	Data collection methods	Blinding	Dropout rate	Grading
Al Badri et al., 2020 [34]	Weak	Longitudinal	Strong	Strong	Moderate	Moderate	Moderate
Dressel et al., 2007 [35]	Moderate	Longitudinal	Moderate	Moderate	Moderate	Strong	Strong
Dressel et al., 2009 [36]	Moderate	Longitudinal	Strong	Strong	Moderate	Weak	Moderate

### Other occupational obstructive lung diseases

Only two studies were specifically dedicated to occupational obstructive pulmonary disease (See Supplementary Table 3, Additional File 3). A large, population study [37] and one from a nanoparticles research team [38].

The first study was conducted on 13,336 subjects from the National Health and Nutrition Examination Survey who underwent FeNO and spirometry measurements. COPD was defined as pre-bronchodilator FEV_1_/FVC < 70%. Occupational exposure to mineral dusts, organic dusts, exhaust fumes, other fumes, and second-hand smoking was significantly correlated with COPD. Long-term occupational exposure to organic dusts, exhaust fumes, and second-hand smoking in the workplace positively correlated with COPD in subjects with FeNO ≤50 ppb. The probably asthmatic group (defined based on a FeNO > 50 ppb) from workplaces with long-term organic dust and exhaust fumes exposure had lower risk for COPD. This would suggest two conclusions: first, that eosinophilic inflammation is less associated with COPD in workplaces with exposure to inhalants and second, that similar long-term exposure might lead to different types of airway inflammation.

The second study focused on chronic bronchitis. This was a small study investigating the personnel with long time exposed to nanoparticles (average + standard deviation = 18 + 10.3 years), in which post shift spirometry, NO, and tumor necrosis factor (TNF), leukotriene B4 (LTB4), leukotriene E4 (LTE4) in exhaled breath condensate were compared to non-exposed (office workers) controls. Chronic bronchitis was identified from anamnesis. Baseline NO was not significantly different between the exposed and non-exposed groups, but reduction in NO, FEV_1_, and FEV_1_/FVC was noticed post shift in the nanoparticles workers. The authors explained the FeNO reduction as an expression of the oxidative mechanisms induced by nanoparticles in the airways which lead to consumption or scavenging of NO [38]. Because there was no chronic bronchitis in the non-exposed group, direct comparisons of FeNO in patients with occupational bronchitis and controls were not performed.

A project conducted in a cohort of diesel engine testers explored the occupational exposure impact on the respiratory system. This project found an obstructive lung pattern to be representative for the long-term effects of these hazards, after adjustment for age, weight, height, smoking, and drinking habit [39]. Moreover, when smokers and non-smokers were compared inside the diesel exhaust group, the lung function was similar, suggesting that occupational exposure had greater effect on lung function than smoking. In the same sample, FeNO was also measured, with no difference between the exposed and the non-exposed subjects [40]. Unfortunately, the relation between FeNO and the diagnose of COPD was not presented. However, there was a significant reduction in FEV_1_ and FEV_1_/FCV, compatible to the ventilatory pattern of occupational bronchitis in the exposed workers; the FeNO was similar to the control group.

Two other studies refer, although not as a main scope, to the relation between FeNO and occupational obstructive lung disease. The first explored several inflammatory markers, such as FeNO, interleukin 8, and nitrite in the exhaled breath condensate in non-smoking employees working in a repeated water damaged building, with improper ventilation and a high level of mould [41]. The exposure started approximatively 5 years before. No relation between current symptoms and FeNO was found. FeNO was significantly lower only in the physician diagnosed chronic bronchitis group. The second study found a significant decrease in FEV_1_ and FEV_1_/FCV in workers exposed for a short time to petrochemical hydrocarbons from oil refineries as compared to a matched group of white collar workers. The samples of this study did not include COPD or asthma patients. FeNO was measured once, during the working hours, and there was no specification of the relation of this measurement to the recent exposure or duration of exposure. Compared to controls, the FeNO mean was lower, but not statistically significant [42], although this study had some uncertainties on the selection procedures (See Supplementary Table 4, Additional File 4). In patients with distal airways obstructive syndrome previously exposed to fiber glass dust, FeNO was not correlated to the cumulative exposure [34] but the alveolar component of the FeNO was not measured. The study was retrospective and the selection of the patients is not clearly stated.

### Interstitial lung disease

FeNO was also a subject of research in interstitial lung diseases (See Supplementary Table 5, Additional File 5 [43–45]). Initial findings in HP showed that the alveolar flux of FeNO was higher than in asthma and in healthy controls [35]. These results were confirmed by other research which highlighted FeNO as a distinctive feature of HP [36]. For this purpose, even a cut-off value of 41 ppb, as optimal sensitivity (76.9%) and specificity (85.4%) to diagnose HP was defined. Unfortunately, none of these studies mentions any data about the occupational exposure.

The two studies on HP which covered also the occupational exposure revealed no signal of FeNO levels in occupational HP. The first [46] compared 11 cases of confirmed HP to 14 cases of suspected HP, which did not meet all major criteria: identification of the exposure and appropriate medical history and/or detection of precipitins in serum or broncho-alveolar lavage, histologic pattern of HP, and SIC positive. FeNO was measured prior and 24 h after SIC. The study showed no difference in the baseline FeNO between the two groups and no difference in the FeNO level before and after SIC in cases confirmed with HP, with positive SIC.

The second study was specifically designed for the investigation of small airway disease in HP [47]. FeNO was measured at baseline and after 4 weeks of treatment. Despite the functional and clinical improvement (reduced symptoms, better 6-minute walk test), FeNO did not change. Data collection and confounders are properly addressed, but there is still some bias in the selection process; they are both cases from reference centers, a bias which can be difficult to surmount for a relatively rare disease, which needs extensive and sophisticated tests for diagnose (See Supplementary Table 6, Additional File 6 [43–45]).

In pneumoconiosis, the utilization of the FeNO was evaluated in a smaller sample of retired coal miners [48] with no clear data on the representativeness. FeNO was significantly lower in current smokers and in those with lower FEV_1_. No differences were noted between patients with small or large opacities and controls (formerly exposed workers without silicosis). Exposure to carbon nanotubes was also related to lower FeNO [49] after adjustment for doctor diagnosed cardiovascular, inflammatory or metabolic disease, educational level, recent infection, white blood count and previous exposure to chemicals. The relation was more robust in nonsmokers and became statistically no significant when corrected for previous exposure to nanoparticles.

On the contrary, in workers with asbestosis and asbestos plaques, FeNO was significantly higher than in controls [50]. However, patients with asbestos-related diffuse pleural thickening had similar FeNO as the controls. An inverse relation between FeNO and total lung capacity was found in cases with asbestosis. The authors suggested a continuation of the inflammation even in quiescent lesions (plaques; in diffuse pleural thickening. In their interpretation, either the process of fibrosis is completed and local inflammation was minimal, or the fusion of visceral and parietal pleural layers altered the NO production in these areas.

FeNO had the tendency to decrease with higher cumulative exposure to beryllium [51], but the differences among high exposed, low exposed and controls were not statistically significant. The number of patients with diffuse interstitial fibrosis was not mentioned in this study, but adjustment to this variable did not influence the FeNO.

## Discussion

Introducing FeNO measurement in clinical practice has many advantages: the method is easy to perform, it is non-invasive, reliable, and does not cause any side effects. The economic cost has already been assessed for introducing it in primary medicine in asthma diagnosis and seems to be promising [52]. Some national asthma guidelines include FeNO in the initial assessment and in the therapeutic approach [53], but for the occupational medicine practice there is no consensus about its usefulness. The variety of exposures and working conditions, on one hand, and of the diverse respiratory pathologies, on the other, represent important barriers, which have to be overcome for a definitive answer.

One objective of this systematic review was to identify possible applications of FeNO measurement in occupational medicine. In brief, for the following occupational related diseases FeNO measurement may be useful: asthma, occupational bronchitis, and interstitial lung diseases.

In OA, FeNO was used for diagnosis, and for proving the effect of an intervention. These were well designed studies, with a comprehensive exposure assessment and high-quality methods for the variables introduced in the analysis, although they came to both consistent and divergent conclusions.

The timing of FeNO measurements after SIC (or after exposure to asthma agents) was 24 h after exposure in 8 of the 13 studies. Some studies measured FeNO earlier and after 24 h, but finally communicated the results of the 24 h measurement, which seems to be the best approach for SIC. The others based their conclusions on the baseline FeNO or on a longer exposure (e.g. 2 weeks). If the latter method was used, the FeNO variation was compared to the ambulatory lung function variation during work and outside the work, and SIC. The expert evaluation of the cases found FeNO measurement valuable for few patients with a SIC negative test, but the sample was quite small.

The results regarding sensitivity and specificity for the included studies are comparable. All studies showed better specificity and negative predictive value by adding FeNO in the diagnosis process. The major disagreement to be addressed by future research is related to the significant level of variation. In this respect, the results were very heterogeneous, even in studies performed in reference centers, in strictly controlled laboratory conditions. The lowest level of variation considered as a good compromise between sensitivity and specificity was 13 ppb, [18], others found 17 ppb [20]; in other studies the variation was expressed as a percentage from the initial level (either 20% or 25% increase) [19, 24]. Differentiation between smokers and nonsmokers is also important, as smoking has a major influence on the FeNO level. Even if smokers have generally lower levels of FeNO, asthmatic smokers have higher FeNO levels than non-asthmatic smokers [54]. In one study included in the current analysis, the significant level for smokers was 14.7 ppb, which was 33% lower than the one for non-smokers [21].

Some studies recognized the value of baseline FeNO and considered as significant the levels generally accepted for this pathology [15], but, for example, in isocyanate exposure the baseline FeNO was significantly higher (62 ppb) in those with positive SIC [28].

Considering all above, we found consistent findings about the time of measurement and that the specificity was higher than the sensitivity of the FeNO variation in SIC. The main argument against the current introduction of FeNO as criteria of diagnosis the occupational asthma is the discrepancy between the significant thresholds detected in different studies.

The conclusion of the studies in which FeNO was used to demonstrate the effectiveness of an intervention is similar. In both projects referring to farmers’ asthma, FeNO proved to be more sensitive than spirometry in measuring the result of an intervention. However, the drop out rate was quite high in the 1 year follow up, which reduces its value for the long term prediction of the symptoms.

In the project dedicated to occupational asthma in farmers [32, 33], FeNO was a more sensitive indicator of short and long-term changes of the bronchial inflammation. Other studies of non-occupational asthma concur to the same conclusion. In suspected patients with suspected asthma and normal FEV_1_ and FEV_1_/FCV, FeNO significantly correlated with the hyperresponsiveness to methacholine [55]. Persistent high FeNO also predicted a more rapid deterioration of the lung function in asthmatics [56].

The value of FeNO in COPD is less well characterized than for asthma. Smoking, a major risk factor for COPD, largely contributes to concealing the NO results. In fact, in COPD patients who stop smoking, FeNO is significantly higher than in those who did not quit [48]. In the stable periods of COPD, FeNO is mildly elevated [50] with no relation to eosinophilic inflammation [48]. A meta-analysis showed that FeNO is not elevated in COPD exacerbations [50]. During exacerbations, the level of FeNO does not depend on the severity of the disease but helps differentiate between asthma and COPD [57] and might be predictive of a better response to corticosteroids [49]. There are arguments from the general population studies to consider FeNO for an early detection of COPD [58] and asthma-COPD overlap, a condition associated with several occupations [59]. Previous international standards for the FeNO technique recommended the single expiratory technique [60], which measures predominantly the larger airways component of NO. The current ERS recommendation [61] explicitly refers to the alveolar component of NO (mainly to the concentration of NO in the gas phase of the alveolar or acinar region) and to the procedures for the evaluation for small aiways inflammation and interstitial diseases using this technique. The technique is more time consuming and implies more sophisticated quality assurance procedures and there are few publications reporting reference values in the general populations.

Because of the above limitations of FeNO, the knowledge regarding values for patients with obstructive lung diseases (apart from asthma) is limited. The largest study [39] which was included in this review underlined the need to continue the investigation in this area, particularly for mineral dusts, organic dusts, exhaust fumes, other fumes, and second-hand smoking. In two other studies [38, 41] FeNO was reduced when measured post-shift in two studies assessing obstructive lung diseases. Both investigations included only nonsmokers with a cumulative exposure for more than 5 years to occupational agents associated with bronchitis. In the group exposed to nanoparticles the post-shift FeNO was significantly reduced [38], while in the group exposed to indoor air pollution a similar impact was noticed only in patients with chronic bronchitis [41]. Both studies included other markers of acute airway inflammation,which were significantly modified during the work shift. It is to be noted that in the first study [38], chronic bronchitis was present only in the exposed group. Even if these two studies provide arguments about the acute effect of exposure, only the second would be of interest for an enhanced post exposure inflammatory response in patients already diagnosed with chronic bronchitis.

The studies conducted in diesel engine exhaust [40] and petrochemical industry [42] measured FeNO only once. There was no difference in results between the exposed and non-exposed persons. But the analysis did not mention whether there were differences among those with and without chronic bronchitis or the timing when the FeNO was measured in relation to the recent exposure.

In view of these findings, measuring post-shift FeNO in workplaces associated with a risk for occupational obstructive lung disease other than asthma could be of interest in confirming the enhanced response to respiratory hazards in work-related chronic bronchitis, although the.. supporting evidence is limited and related only to some occupational exposures. Measuring the alveolar FeNO could further clarify this relation in the future.

Interstitial lung diseases (ILD) include a large group of diffuse parenchymal disorders, with a spontaneous evolution towards fibrosis. The need for an early diagnosis is crucial [62], particularly for the occupational diseases in which cessation of exposure could prevent the evolution. The initial positive ability of FeNO to discriminate among different types of ILD, was contradicted in more recent publications [63]. However, if the two compartment models (bronchial and alveolar) of FeNO are calculated [64], the alveolar NO becomes relevant [65].

Two studies investigated FeNO in occupational HP but did not find any added value from this measurement. Neither of these made the distinction between bronchial and alveolar FeNO, as was previously reported to be characteristic for the extrinsic alveolitis [35]. Few other studies concerned exposure to silica and asbestos: in silicosis no difference was reported between simple and complicated silicosis. After asbestos exposure, only asbestos plaques were related to a higher FeNO, while the supposed explanation for this finding remains speculative.

Based on the results of these studies, FeNO has a marginal role, if any in the diagnostic of occupational ILD. Biologically, the alveolar component could be of interest, but was not investigated.

## Conclusions

There is an extensive literature on the utilization of FeNO in occupational medicine.

In occupational asthma, FeNO can be used as a marker of inflmmation because of its correlation with the hyperresponsiveness to methacholine and persistent high FeNO also predicted a more rapid deterioration of th lung function in asthmatics. Despite this, there is no consensus on the significant value for diagnosis, or on the magnitude of the change of FeNO level after exposure. There is some consensus about the optimal time to measure FeNO after exposure, mainly after 24 h. FeNO could to be more sensitive than spirometry in measuring the result of an intervention in OA.

With regards to other respiratory diseases, the number of studies is limited and the results are inconsistent. If there is a role in other occupational obstructive respiratory diseases, current data suggest to perform the measurement after the work shift to assess the occupational obstructive respiratory diseases. For both occupational bronchitis and interstitial lung disease, the evaluation of the alveolar NO component is probably the most suitable.

## Supplementary Information

**Additional file 1.**

**Additional file 2.**

**Additional file 3.**

**Additional file 4.**

**Additional file 5.**

**Additional file 6.**

## Data Availability

NA

## Abbreviations

BMI: Body mass index
COPD: Chronic obstructive lung disease
EPHPP: Effective Public Health Practice Project
FeNO: Fractional exhaled nitric oxide
FEV_1_: Forced expiratory volume in the first second
FVC: Forced vital capacity
GINA: Global Initiative for Asthma
HMW: High molecular weight
HP: O hypersensitivity pneumonitis
LMW: Low molecular weight
LTB4: Leukotriene B4
LTE4: Leukotriene E4
NO: Nitric oxide NO
OA: Occupational asthma
PEF: Peak expiratory flow
SIC: Specific inhalation challenge test
TNF: Tumor necrosis factor
